# Seeking motivation for selecting Medical Profession as a Career Choice

**DOI:** 10.12669/pjms.36.5.2799

**Published:** 2020

**Authors:** Madeeha Hassan, Fatima Shahzad, S H Waqar

**Affiliations:** 1 Madeeha Hassan, MBBS. Postgraduate Resident, Department of Medicine, Pakistan Institute of Medical Sciences, Islamabad, Pakistan; 2 Miss Fatima Shahzad, Undergraduate Medical Student. Al-Nafees Medical College, Islamabad, Pakistan; 3 Prof. S H Waqar, MBBS, FCPS (Surgery), FICS, MCPS-HPE Professor and Head Department of Surgery, Pakistan Institute of Medical Sciences, Islamabad, Pakistan

**Keywords:** Career choice, Medical student, Motivation

## Abstract

**Objective:**

To determine the motivational factors of medical students for selecting medical career as a profession.

**Methods:**

This was a survey based cross-sectional study. Medical students of first and second year from private and public medical colleges of Rawalpindi and Islamabad were recruited via convenient sampling and a self-based questionnaire was distributed to them. Besides demographics, questions were based on factors influencing medical students to pursue medical career. Data was collected and descriptive analysis was done using SPSS 21.

**Results:**

A total of 300 medical students participated in the study; 129 (43%) of them were males and 171 (57%) were females with mean age of 19.35 years. Among Humanitarian based questions; Serving Humanity gained highest percentage, followed by sympathies for mankind and health for everyone sequentially. In Societal factors, Prestigious Profession and in Scientific factors based questions Challenging Field ranked first. Amongst all the questions Prestigious Profession takes the lead.

**Conclusion:**

Major motivation behind selecting medical field was prestigious profession followed by altruism. By understanding medical students’ motivational factors for pursuing medical field we would be able to analyze the future trend of professionals.

## INTRODUCTION

Motivation is a psychological persuasion that involves a person’s eagerness or enthusiasm to endeavor a set goal at an expense of hard work and sacrifices. It is a prime mover of human behavior, a dynamic element^1^, a critical component of learning^2^,^3^, a psychological process that describes person’s willingness to use the best of his abilities to achieve a single aim.^1^ Thus, motivation is a determining factor, an inner driving force of an individual to accomplish personal or organizational objective. On the whole, motivation is considered as a process which is evoked by a pertinent stimulus leading to extraordinary ability to achieve a goal.^3^

Thousands of people set their goal of life to become a doctor. However, the daunting experience of night shifts and grueling hours of practice makes it not an easy goal to achieve.^4^ Motives and beliefs of medical students help accept the grueling training program easily.^1^ Passion, motivation and professional expertise are a long suit for exceptional performance in a diverse field like medicine.^5^ The ascendary of medical profession over other professions has made it the most wanted career choice.^6^ Students’ attraction towards medical field depends upon many factors including its needfulness, social and financial status and altruism.^6^-^8^ For healthcare workforce altruistic approach is still crucial but not the only driving force, controllable lifestyle such as career opportunities, salary package and workplace are equally important.^4^

For a medical student, the motivation is regarded as readiness to pursue medical training regardless of burnouts or delays or impediment to professional progress.^6^ Thus, a right career choice is a major factor in deciding a promising future. However, due to lack of professional career counseling after F.Sc. or A-levels, high achievers are usually attracted towards medical profession due to its social repute, high income incentives and lack of knowledge about other fields.^8^ Moreover, as world is facing a critical scarcity of medical work force,^4^ the doctor to patient ratio is notably distributed in medical field.^9^ The limited manpower in doctor’s community has led to serious work force crisis.^9^

Thus, it would be a baseline study to explore the driving force for motivating students to pursue medical profession and their notion for further contribution in medical field advancement. This study will help evaluate the initial drive to choose a particular profession and understanding of perception to effectively utilize human resources.

## METHODS

A cross-sectional descriptive survey was conducted. Convenient sampling was done by distributing self-prepared questionnaire along with attached consent forms, later removed, to ensure anonymity, to medical students of first and second year only from private and public medical colleges of Rawalpindi and Islamabad. They were selected because usually students in initial years have not been exposed to clinical environment and have higher achievement motivation not merely, influenced by the environment. Sample size was 300, calculated by WHO sample size calculator.

After a detailed literature review, a pre-tested semi-structured self-administered questionnaire was designed based on previous similar studies. The practically relevant and important items were selected by the investigators to prepare the preliminary questionnaire. The questions were then reviewed and revised as necessary for accuracy and grammar. The pre-final version of the questionnaire was pilot tested on students. They were not included in the final study. Following pretesting, finer corrections were made to improve the clarity so that the intended meaning was retained.

Brief demographic details about participants including age, gender, and parents’ educational status were questioned. Questionnaire had items based on factors influencing medical students to pursue medical career. Three items were descriptive type and remaining all were Likert type questions with responses given from 1. Strongly Disagree to 5. Strongly Agree. Statistical analysis was done using SPSS 21. Frequencies, percentages, mean and standard deviation were calculated.

### Ethical approval

This study was conducted after approval from the Ethical Review Board of Shaheed Zulfiqar Ali Bhutto Medical University, Islamabad, (Ref No. F.1-1/2015/ERB/SZABMU/03/7, dated April 7, 2020).

## RESULTS

A total of 300 medical students took part in the study; 129 (43%) of them were males and 171 (57%) were females, mean age was 19.3+1.1 years. The decision of pursuing medicine as a career for 194 (64.4%) students was their own, 78 (26%) joined medical profession on parents’ will. However, for 28 (9.3%) respondents, it was a by chance decision.

**Fig. 1:**
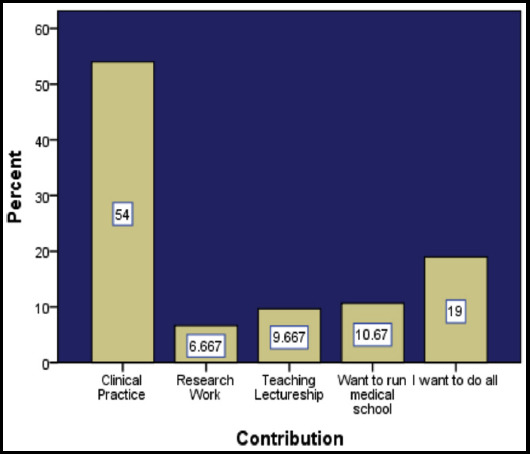
Ways of contributing in medical field.

On inquiring how would they prefer to contribute in the field of medicine, a variety of options was reported by students. Fig.1 illustrates how our respondents want to contribute in medical field.

Rest of the questions were based on humanitarian, societal and social factors and scaled according to likert-type. Among humanitarian based questions; serving humanity (mean 3.72±0.066) gained highest rank, followed by sympathies for mankind (mean 3.54±0.062) and health for everyone (mean 3.51±0.057) sequentially. In societal factors, prestigious profession (mean 3.84±0.058) and in scientific factors based questions challenging field (mean 3.45±0.057) was ranked first. Amongst all the questions prestigious profession takes the lead.

Mean and standard deviation of each item was calculated for both genders separately. The response of difference of opinion with respect to gender difference is shown in Table-I.

**Table-I:** Response to items W.R.T gender difference.

Items	Gender	Mean	Std. Deviation
Serving Humanity	Male	3.74	1.194
	Female	3.71	1.115
Sympathies & Empathies to deprived ones	Male	3.67	1.026
	Female	3.44	1.091
Medicine, a science based profession	Male	3.53	1.024
	Female	3.09	0.993
High Income Profession	Male	3.43	1.088
	Female	3.08	1.046
High Social Standards	Male	3.53	0.953
	Female	3.42	0.956
Prestigious Repute	Male	3.89	0.937
	Female	3.80	1.046
Challenging Field	Male	3.46	1.061
	Female	3.44	0.927
Research Oriented	Male	3.27	0.974
	Female	3.05	0.903
Patriotic Spirit motivated me	Male	3.25	1.153
	Female	3.04	0.910
Lack of facilities influenced me	Male	3.18	1.128
	Female	2.92	1.071
Witnessed a disaster	Male	2.88	1.122
	Female	2.79	1.123
Woeful Happenings made me choose medicine	Male	3.31	1.080
	Female	3.05	1.142
Sufferings of near ones motivated me	Male	2.98	1.114
	Female	2.85	1.237
My sufferings motivated me	Male	2.62	1.112
	Female	2.49	1.210
I wanted health for everyone	Male	3.47	0.884
	Female	3.54	1.047
Wanted to fight against Endemic disease	Male	3.52	0.936
	Female	3.16	1.150
I had a role model who influenced me	Male	2.98	1.259
	Female	2.96	1.229

## DISCUSSION

It requires drive from within to achieve a goal.^9^ The difficult the task, the greater the motivation is required. Motivation positively influences academic and practical performance and enables a person to deal with hardships of career attainment.^2^,^3^ Hence, it is important to understand the prime movers that influence students to choose medical career. In our study, prestige was the most reported prime mover with a mean of 3.84±0.05 followed by altruism (mean 3.7±0.66). A study conducted in Egypt by Kabil NS et al.,^10^ ranked prestige as the most highly considered motivational factor followed by financial security for choosing medical career. In an Indian study by Narayanasamy et al.,^1^ altruism was the most customary motivational factor among medical students (mean 4.26±0.9). Likewise, a Nepal based study by Torres-Roman et al.,^11^ reported altruism followed by prestige as major motivation in medical career selection. These motivational factors may vary geographically owing to difference in cultural and professional aspects of that country.

Our 64.6% students joined medical profession by their own will, 26% were influenced by their parents will and 8% selected medicine because of lack of knowhow about other fields. Same result in depicted in Kabil NS et al.,^10^ study, reason for pursuing medicine for 67% respondents was their own interest and for 20% was parents influence. Furthermore, Kabil NS et al.,^10^ concluded that males are more inclined towards prestige and high income, however, our study didn’t support opinion difference between two genders.

They say it’s the positives that keep us going. There are examples when the most debilitating fatigues come out as the most positive driving force. Our respondents with a mean of 2.9±0.06 agreed that they witnessed near ones suffering from debilitating disease and a number of students (mean 3±0.06) revealed that deprivation of basic necessities provoked them to study medicine. Findings of study by Gyorffy et al.^12^, revealed 13.5% witnessed near ones suffering and 39% respondents were deprived ones.

In response to serve their own native areas despite lack of facilities, only 12% of our respondents show willingness to work. This is due to the fact that the working environment, basic infrastructure, good accommodation and privacy are very crucial for a person being posted at a work place.^13^ Since our basic health system lacks such facilities therefore, only a handful of doctors are willing to work at their native places. Thus, good working environment came out to be an important driving force for our respondents.

Research provides a building block for medical headway. The role of medical students in research involvement is essential for medical advancement. Research positively provokes analytical thinking and has a positive impact on career choice in medical students.^14^ Unfortunately, little drift and interest is shown towards it mainly due to grueling hours of clinical practice, tough medical studies and little access to resources and lack of research mentors.^14^ Only 6.6% of respondents agreed to pursue research in their future career. This is probably due to lack of research orientation at undergraduate level. Therefore, in our study, research had a lower magnitude of impact on career selection.

### Limitations of the study

It include a small sample size. Secondly, our study population belongs to higher or higher-middle class families, hence, viewpoints of lower middle class families could not be attained that might limit generalizability of our results. The study could compare attitudes of medical students on parental medical and non-medical background basis; however, it was not questioned at all.

## CONCLUSION

Major motivation behind selecting medical field as a career choice was prestigious profession followed by altruism. However, factors related to professionalism and economic security have also influenced students to persue medicine as a career. By understanding medical students’ motivational factors, we would be able to analyze the future trend of professionals. Efforts should be made by professional medical institutes to help medical students preserve their motivations thus ultimately helping them to be a good clinician and be productive for their society.

### Recommendation

As suggestion, a multi-method programatic pre-selection criteria is recommended permitting only those applicants who possess right cognitive and non-cognitive approach to be a competent student and a conscientious doctor. This study may aid professional development of medical educators. Medical education departments can set their stratagem and systematize appropriate professional teaching skills that suit learner’s attitude. And finally, in the light of student’s economic expectations from the medical field authorities should pay handsome incentives to stop brain drain.

### Authors’ Contribution`s

**MH** did data collection, statistical analysis and manuscript writing, and she is responsible and accountable of the study.

**FS** did data collection and manuscript writing.

**SHW** conceptualized, edited, reviewed and finally approved the manuscript.
